# Device-measured physical activity and incident affective disorders

**DOI:** 10.1186/s12916-022-02484-0

**Published:** 2022-09-06

**Authors:** Frederick K. Ho, Fanny Petermann-Rocha, Solange Parra-Soto, Jirapitcha Boonpor, Jason M. R. Gill, Stuart R. Gray, Jill P. Pell, Carlos Celis-Morales

**Affiliations:** 1 Institute of Health and Wellbeing, University of Glasgow, Glasgow, UK; 2 Institute of Cardiovascular & Medical Sciences, University of Glasgow, Glasgow, G12 8TA UK; 3 Facultad de Medicina, Universidad Diego Portales, Santiago, Chile; 4 Human Performance Lab, Education, Physical Activity and Health Research Unit, University Católica del Maule, 3466706 Talca, Chile

**Keywords:** Accelerometery, Physical activity, Depression, Anxiety, Mental health

## Abstract

**Background:**

Studies on physical activity (PA) and mental health are largely limited to self-reported PA. This study aims to use prospective cohort data to investigate the association between device-measured PA and affective disorders.

**Methods:**

A total of 37,327 participants from UK Biobank who had not had any prior affective disorder diagnoses were included in this prospective cohort study. Wrist-worn accelerometers were used to measure total, light (LPA), moderate (MPA), and vigorous (VPA) PA. Associations between PA domains and affective disorders were analysed using penalised splines in Cox proportional hazard models. Analyses were adjusted for other intensity-specific PA and sociodemographic and lifestyle factors. Sensitivity analyses were conducted adjusting for body mass index and longstanding illnesses as well as excluding events in the first 2 years of follow-up. Preventable fractions for the population were estimated for MPA and VPA.

**Results:**

Over a median follow-up of 6.8 years, 1262 (3.4%) individuals were diagnosed with affective disorders. Replacing 30 min of sedentary behaviour in a week with MPA (HR 0.95, 95% CI 0.94–0.97) or VPA (HR 0.91, 95% CI 0.85–0.98) was associated with lower risk of affective behaviours, up to 500 and 120 min of MPA and VPA. Assuming causality, 5.14% and 18.88% of affective disorders could have been prevented if MPA ≥150 min/week and VPA ≥75 min/week were achieved, respectively, across the study population.

**Conclusions:**

Device-measured MPA and VPA were associated with lower risk of affective disorders. The potential mental health benefits of MPA continue to accrue above the current World Health Organization recommendation.

**Supplementary Information:**

The online version contains supplementary material available at 10.1186/s12916-022-02484-0.

## Background

Psychiatric disorders are one of the top contributors to the global burden of diseases accounted for 14% of total years living with disability in 2017 [1]. Among psychiatric disorders, affective disorders are the most common type. Current treatment for affective disorders includes medications, such as selective serotonin reuptake inhibitors [2], psychotherapy [3], or even exercise on prescription [4]. However, medications often have various side effects [5] and cognitive behavioural therapy is only moderately effective [3]. In view of these, identifying strategies to prevent affective disorders is important to reduce their public health burden.

One potential modifiable factor for affective disorders is physical activity (PA). Meta-analyses of prospective studies have shown that PA was associated with lower risk of depression [6] (OR = 0.78) and anxiety [7] (OR = 0.66). However, the meta-analyses also acknowledged multiple limitations of existing studies: most notably, the reliance on self-reported measurement and the failure to adjust for key confounders, such as ethnicity and chronic conditions [6, 7]. Inconsistency between self-reported and objectively measured PA has been well reported which could substantially distort effect sizes [8]. This issue could be further amplified for affective disorders since the inaccuracy in self-report could be dependent on mental health.

There were experimental evidence supporting the causal effect of PA on affective disorders and general mental health, but most trials compared PA intervention with usual care [9, 10]. One important question that could not be easily answered by trials is the dose-response association between PA and mental health outcomes, as well as whether this depends on the intensity of PA. Focusing on duration, a cross-sectional study of 1.2 million participants reported a U-shaped relationship between PA and mental health, whereby people who self-reported to perform moderate-duration of PA had fewest mental health symptoms [11]. However, as with most previous studies, such findings could be due to bidirectionality or recall bias [12].

The current study aimed to determine whether, and to what extent, device-measured PA was associated with affective disorders, including depression and anxiety. This study also investigated whether there was any dose-response relationship in regard to PA intensity and explored its public health implications by estimating the preventable fractions for the population (PFPs).

## Methods

The UK Biobank cohort study enrolled over 500,000 participants aged 37–73 years at baseline from the general population (5.5% response rate) [13]. In brief, between 2006 and 2010, participants attended one of 22 assessment centres across Scotland, England, and Wales [14, 15]. All participants completed a touch-screen questionnaire, had physical measurements taken, and provided blood, urine, and saliva samples at baseline. Among all UK Biobank participants, about half had linked primary care data and were included in this study (Additional file 1: Fig. S1).

### Device-measured PA

Axivity AX3 wrist-worn triaxial accelerometers were used to collect objective PA measurements from 103,686 UK Biobank participants between 2013 and 2015. The dominant wrist of each individual was used over a period of 7 days at 100 Hz and combined into 5-s epochs for analysis [16]. The 7161 participants with insufficient wear time (<72-h wear), missing data, or poor device calibration were excluded, leaving 96,525 participants with valid device-measured PA data. The median wear time was 6.91 days with less than 20% of participants wore < 6 days. More details about data collection and processing can be found elsewhere [16].

Minutes per week (min/week) of light (LPA), moderate (MPA), and vigorous (VPA) PA were determined as the time spent between >30 milligravities (*mg*) and 125 *mg*, >125 *mg* and 400 *mg*, and >400 *mg*, respectively [17, 18], extrapolated from fraction of time spent over the total wear time. This assumes the time spent in various PA was similar in measured and unmeasured period. Total PA was expressed as total metabolic equivalent of task (MET)-minutes/week accounting for both intensity and duration and was calculated as time spent in LPA × 2 + MPA × 4 + VPA × 8, adapting the International Physical Activity Questionnaire scoring recommendation [19].

### Affective disorders

Incident affective disorders were extracted from both hospital and primary care records to ensure comprehensive ascertainment. Dates and causes of hospital admissions were identified via record linkage to Health Episode Statistics (England and Wales) and the Scottish Morbidity Records (Scotland). Details of the linkage procedure can be found at http://content.digital.nhs.uk/services. The start of follow-up was the date where all device-measured PA measurements were completed. Participants with affective disorders prior to that date, based on retrospective record linkage, were excluded from the analyses (Additional file 1: Fig. S1). Retrospective linked data dated back to 2004 were used as the Quality and Outcomes Framework in primary care practices that were implemented that year. Hospital admission data were available until September 2021 in England, July 2021 in Scotland, and February 2018 in Wales. Using the International Classification of Diseases, 10th revision (ICD-10), affective disorders were defined as F30-F39 and F40-F44, depression as F32-33, and anxiety as F40-44.

### Adjustment variables

Age, when PA data were collected, was determined from dates of birth and PA assessment. Ethnicity was self-reported and categorised into White, South Asian, Black, Chinese, mixed ethnic background, and others. Area-based deprivation was derived from postcode of residence, using the Townsend score [20]. Educational attainment was based on self-report of the highest level of qualification. Sleep duration was self-reported in hours as there were no reliable objectively measured data. Smoking status was self-reported and categorised as never, former, or current smoker. Alcohol consumption was calculated based on self-reported frequency and volume of drinking. Dietary intake of fruit and vegetables, red meat, processed meat, and oily fish was self-reported. Height and body weight were measured by trained nurses during the baseline assessment. Body mass index (BMI) was calculated as (weight in kg)/(height in m)^2^. Longstanding illnesses were self-reported as any longstanding illness, disability, or infirmary.

### Statistical analyses

Descriptive characteristics by quartile of total PA are presented as means with standard deviation (SD) for continuous variables and frequencies with percentages for categorical variables.

Nonlinear associations between PA and incident affective disorders were investigated using penalised cubic splines fitted in Cox proportional hazard models. The penalised spline is chosen as it is not sensitive to knot numbers and placements [21]. In these analyses, PA variables were truncated to be between 2000 and 12,000 MET-min/week for total PA, 300 and 3600 min/week for MPA, 0 and 1800 min/week for MPA, and 0 and 240 min/week for VPA to ensure the reliability at the tail end of the splines. These truncation thresholds were roughly the 1st and 99th percentile for each of the variables. The reference points were the minimum of the truncated PA variables. An analysis assuming linearity was performed in the range where the associations appeared linear to extract easily interpreted estimates. This was replicated to use MET-minutes/week instead of time spent. Analyses were adjusted for these confounders: age, sex, ethnicity, deprivation, education, sleep duration, smoking, alcohol intake, and dietary intake based on the causal hypothesis shown in Additional file 1: Fig. S1. For analysis of LPA, MPA, and VPA, the three PA variables were mutually adjusted. Because LPA, MPA, VPA, and sleep duration were all included in the model, the hazard ratios of PA could be interpreted as replacing the time spent on sedentary behaviour by that PA [22].

To illustrate the joint association of MPA and VPA with affective disorders, a risk matrix was constructed using the categorised MPA and VPA variables: 0–<150, 150–<300, 300–<600, and ≥600 min for MPA and 0–<15, 15–<45, 45–<75, and ≥75 min for VPA. These cut-offs were chosen based on the current WHO PA recommendations [23] and the shapes of associations, as well as the distributions. The least active was used as the reference groups.

Furthermore, three sensitivity analyses were performed. Firstly, PA were not mutually adjusted as LPA and MPA (*r* = 0.42) and MPA and VPA (*r* = 0.49) were moderately correlated which may lead to unstable HRs. Secondly, BMI and longstanding illnesses reported at baseline were additionally adjusted as these could limit the ability of participants to perform PA, but could also be mediators (Additional file 1: Fig. S1). Thirdly, a 2-year landmark analysis was conducted excluding participants who developed affective disorders in the first 2 years of follow-up (*n* = 795), in order to minimise the risk of reverse causation due to sub/pre-clinical mental health issues at baseline.

Preventable fractions for the population (PFPs) [24] were estimated to estimate the proportions of all incident affective disorders that could have been prevented if the individuals in a specific PA categories were as active as the most active group, assuming that the association was causal. It should be noted that this study could not ascertain causality and this analysis only illustrates the relative importance of intensity-specific PA for the study population. All analyses were conducted using the ‘survival’ packages in R 4.2.0. The proportional hazard assumption was checked using Schoenfeld residuals. A *P*-value below 0.05 was considered statistically significant.

### Ethical approval

UK Biobank was approved by the North-West Multi-Centre Research Ethics Committee (Ref: 11/NW/0382). Written informed consent was obtained from all participants.

## Results

Of the 502,459 UK Biobank participants, 96,519 had valid device-measured PA data (Additional file 1: Fig. S2). Of these, 53,480 were excluded because no primary care data were available. There were no significant differences in demographics and device-measured PA (e.g. average acceleration 27.89 vs. 27.90 *mg*) between participants with and without linked primary care data. In the overall analysis of affective disorders, an additional 5712 were excluded due to prior affective disorder diagnoses, resulting in an effective sample size of 37,327. The median (interquartile range [IQR]) follow-up period was 6.8 (6.3–7.4) years. The overall incidence rates for overall affective disorders, depression, and anxiety were 50.8, 25.5, and 30.8 per 10,000 person-years, respectively.

Participant characteristics by device-measured total PA are shown in Table 1. Participants who undertook more PA were generally younger, less deprived, more likely to be female and White, less likely to smoke, and consumed more fruit and vegetables and less red and processed meat. They also had lower BMI and were less likely to have longstanding illnesses. Participant characteristics by LPA, MPA, and VPA are shown in Additional file 1: Tables S1-S3, respectively.

**Table 1:** Participant characteristics

	Overall	Device-measured total PA, MET-minutes/week			
		≤5121	>5121 to 6189	>6189 to 7318	>7318
Total *n*	37,237	9108	9337	9428	9364
Age, years, mean (SD)	56.41 (7.76)	58.65 (7.38)	56.98 (7.67)	55.76 (7.70)	54.32 (7.64)
Male	16,907 (45.4)	4999 (54.9)	4264 (45.7)	4020 (42.6)	3624 (38.7)
Non-White ethnicity	1042 ( 2.8)	244 ( 2.7)	242 ( 2.6)	246 ( 2.6)	310 ( 3.3)
Deprivation index, mean (SD)	−1.84 (2.71)	−1.65 (2.83)	−1.88 (2.69)	−1.95 (2.63)	−1.86 (2.68)
College or university degree	16,103 (43.2)	3715 (40.8)	4159 (44.5)	4226 (44.8)	4003 (42.7)
PA volume^a^, *mg*, mean (SD)	28.00 (7.82)	19.07 (3.10)	24.97 (2.24)	29.73 (2.96)	37.98 (5.51)
LPA, minutes/week, mean (SD)	2052.94 (434.05)	1588.98 (272.07)	1955.66 (248.11)	2185.81 (284.27)	2467.46 (355.04)
MPA, minutes/week, mean (SD)	483.34 (233.67)	251.44 (102.00)	395.58 (105.36)	520.75 (122.31)	758.76 (208.86)
VPA, minutes/week, mean (SD)	31.48 (37.79)	10.96 (15.23)	21.56 (23.48)	33.11 (32.28)	59.70 (50.47)
Sleep duration, hours/day, mean (SD)	7.18 (0.96)	7.24 (1.04)	7.19 (0.95)	7.17 (0.93)	7.12 (0.90)
Smoking					
Never	21,642 (58.1)	4886 (53.6)	5448 (58.3)	5610 (59.5)	5698 (60.9)
Previous	13,237 (35.5)	3469 (38.1)	3317 (35.5)	3301 (35.0)	3150 (33.6)
Current	2358 (6.3)	753 (8.3)	572 (6.1)	517 (5.5)	516 (5.5)
Alcohol intake, units/week, mean (SD)	15.98 (16.00)	16.24 (17.11)	16.22 (16.30)	15.80 (15.14)	15.65 (15.42)
Fruits/vegetable intake, portions/week, mean (SD)	4.21 (2.25)	3.93 (2.16)	4.10 (2.14)	4.25 (2.23)	4.53 (2.41)
Red meat intake, portions/week, mean (SD)	2.06 (1.38)	2.18 (1.40)	2.09 (1.38)	2.02 (1.34)	1.95 (1.38)
Processed meat intake, times/week, mean (SD)	2.80 (1.05)	2.91 (1.05)	2.81 (1.04)	2.77 (1.05)	2.71 (1.07)
Oily fish intake, times/week, mean (SD)	2.66 (0.90)	2.65 (0.89)	2.67 (0.90)	2.66 (0.88)	2.65 (0.91)
BMI, kg/m^2^, mean (SD)	26.70 (4.44)	28.37 (5.04)	26.94 (4.27)	26.25 (4.00)	25.28 (3.79)
Longstanding illnesses	10,137 (27.2)	3359 (36.9)	2610 (28.0)	2203 (23.4)	1965 (21.0)

*MET* metabolic equivalent of task

^a^PA volume as the average acceleration in milligravities (*mg*)

**Fig. 1:**
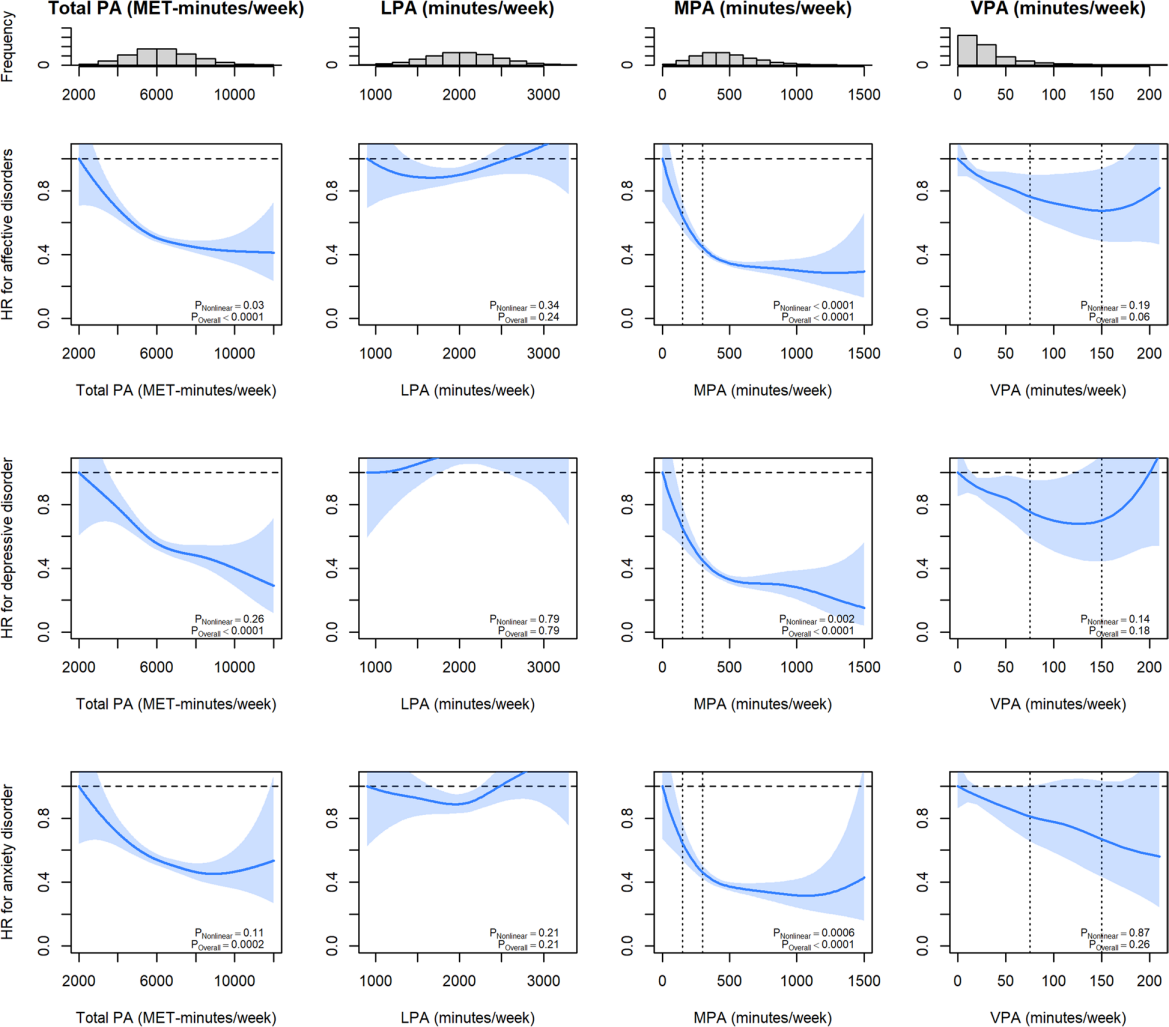
Associations between device-measured PA and affective disorders. LPA, MPA, and VPA were mutually adjusted; all adjusted for age, sex, ethnicity, education, deprivation index, sleep duration, smoking, alcohol intake, and dietary intake of fruits/vegetables, red meat, processed meat, and oily fish. Vertical dashed lines represent current WHO recommendations. LPA light-intensity PA, MPA moderate-intensity PA, VPA vigorous-intensity PA

Total PA, and MPA VPA were associated with lower risk of incident affective disorders when adjusted for sociodemographic and lifestyle (Fig. 1) factors. Compared with the least active participants, performing PA was associated with reduced risk of affective disorders up to 6000 MET-minutes/week, after which the association plateaued. Participants who replaced sedentary behaviours with 150–300 min/week of MPA (HR 0.53 95% CI 0.48–0.58) or 75–150 min/week of VPA (HR 0.71, 95% CI 0.56–0.91) were at lower risk of affective disorders. The association of MPA with affective disorders was broadly linear until 500 min/week and then plateaued. That of VPA was J-shaped with the lowest risk at 120 min of VPA. The associations of MPA with depression and anxiety were broadly similar to total PA, but that of VPA with depression was more U-shaped and that with anxiety was more linear. There were no significant associations for LPA.

When intensity-specific PA were not mutually adjusted, the association of MPA remained similar but those of VPA were less J-shaped and were significant for all three outcomes (Additional file 1: Fig. S3). However, LPA was still not significantly associated with all of the outcomes. When BMI and longstanding illnesses were also added to the models (Additional file 1: Fig. S4), the associations were attenuated but still significant for MPA. Similar results were found in the 2-year landmark analyses (Additional file 1: Fig. S5).

Since the associations were broadly linear up to 500 and 120 min/week of MPA and VPA as shown in the splines in Fig. 1, analysis assuming linearity was conducted for these ranges (Table 2). Replacing 30 min/week of sedentary behaviour with MPA and VPA was associated with 5% (HR 0.95, 95% CI 0.94–0.97) and 9% (HR 0.91, 95% CI 0.85–0.98) lower risk of affective disorders, respectively. The results were similar when PA was adjusted for intensity. When intensity PA were not mutually adjusted, the association of MPA was similar but that of VPA was noticeably stronger.

**Table 2:** Linear association between device-measured PA and affective disorders

	Applicable range (minutes/week)	Per 30 min/week	Per 500 MET-minutes/week	
		HR (95% CI)	HR (95% CI)	***P***-values
**Intensity-specific PA mutually adjusted**				
MPA	<500	0.95 (0.94–0.97)	0.82 (0.77–0.88)	< 0.0001
VPA	<120	0.91 (0.85–0.98)	0.83 (0.71–0.96)	0.01
**Intensity-specific PA not mutually adjusted**				
MPA	<500	0.95 (0.93–0.96)	0.80 (0.75–0.84)	< 0.0001
VPA	<120	0.83 (0.78–0.89)	0.68 (0.59–0.78)	< 0.0001

All adjusted for age, sex, ethnicity, education, deprivation index, sleep duration, smoking, alcohol intake, dietary intake of fruits/vegetables, red meat, processed meat, and oily fish

*MPA* moderate-intensity PA, *VPA* vigorous-intensity PA

**Fig. 2:**
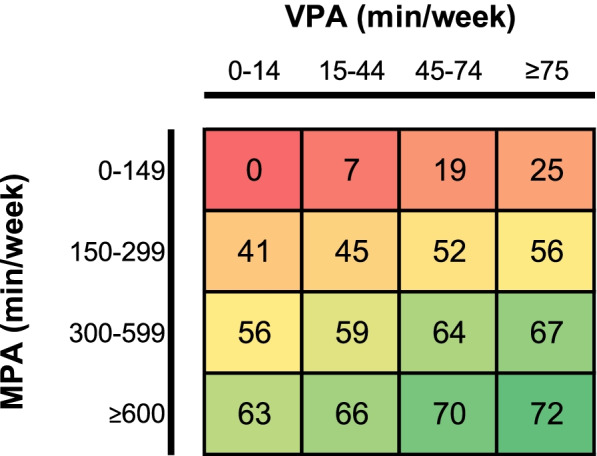
Risk matrix on the joint association of MPA and VPA with affective disorders. Risk matrix is for visualisation purpose only and there were considerable overlaps in the 95% CIs as shown in Table 3. Estimated in Cox regression adjusted for age, sex, ethnicity, education, deprivation index, sleep duration, smoking, alcohol intake, dietary intake of fruits/vegetables, red meat, processed meat, and oily fish. Numbers presented are the associated reduction in hazard (%) compared with the least active group

The risk matrix of the joint associations between MPA and VPA and affective disorders is shown in Fig. 2. LPA was not included since it was generally not associated with affective disorders even when not adjusted for other intensity-specific PA. Assuming causality, a sedentary individual with no MPA or VPA would have 41% risk reduction in affective disorder if they replace 150–299 min of sedentary time in a week with MPA. If that individual further replaces ≥75 min of sedentary time in a week with VPA, their risk reduction would be 56%.

Table 3 shows the proportions of preventable affective disorders. Assuming the associations to be causal, almost one-fourth of incident affective disorders in the study population were attributed to <150 min/week of MPA (5.14%) and <75 min/week of VPA (18.88%). The proportion attributable to MPA was small because only 4% of the study participants performed <150 min of MPA. Conversely, over 90% of participants performed <75 min of VPA, resulting in a larger number of cases attributed to VPA. However, a total of 20.98% of the cases could have been prevented if all participants could perform ≥600 min of MPA a week.

**Table 3:** Proportions of affective disorders attributable to different levels of PA

	Prevalence in study sample (%)	HR (95% CI)	PFP^**a**^	
			% (95% CI)	Cumulative % (95% CI)
**LPA, min/week**				
300 to <1200	2.3	1 (reference)	-	-
1200 to <1500	7.3	0.94 (0.63–1.38)	-	-
1500 to <2000	36.4	1.10 (0.77–1.56)	-	-
≥2000	54.0	1.18 (0.82–1.69)	-	-
**MPA, min/week**				
0 to <150	4.0	1 (reference)	5.14 (4.21–6.10)	5.14 (4.21–6.10)
150 to <300	17.7	0.60 (0.47–0.77)	8.20 (5.49–10.89)	13.34 (10.60–16.41)
300 to <600	51.5	0.45 (0.35–0.58)	7.65 (1.77–14.06)	20.98 (15.70–27.08)
≥600	26.8	0.38 (0.28–0.51)	Reference	Reference
**VPA, min/week**				
0 to <15	42.6	1 (reference)	11.05 (3.42–19.64)	11.05 (3.42–19.64)
15 to <45	36.6	0.94 (0.82–1.08)	7.11 (0.81–13.88)	18.17 (9.66–28.41)
45 to <75	11.0	0.82 (0.65–1.03)	0.71 (−1.77, 3.42)	18.88 (10.36–29.32)
≥75	9.8	0.76 (0.59–0.98)	Reference	Reference

LPA, MPA, and VPA were mutually adjusted; all adjusted for age, sex, ethnicity, education, deprivation index, sleep duration, smoking, alcohol intake, dietary intake of fruits/vegetables, red meat, processed meat, and oily fish

*HR* hazard ratio, *MPA* moderate-intensity PA, *VPA* vigorous-intensity PA, *PPF* preventable fraction of the population

^a^PFP estimated the fractions of all incident affective disorders that could have been prevented if the individuals in those PA categories were as active as the reference group

## Discussion

This study provided robust evidence of an association between device-measured PA and affective disorders, including depression and anxiety. A higher level of PA was associated with lower risk of affective disorders up to 500 min of MPA and 120 min of VPA per week. At an equivalent amount of time, VPA was associated with lower risk of affective disorders than MPA. However, consistent with the literature [25], the potential benefits of MPA continue to accrue after achieving the current PA recommendations (Fig. 2). A larger proportion of population-level mental health burden was attributed to low VPA because a substantial amount of people did not perform sufficient VPA (Table 3).

This study has several important strengths. First and foremost, it utilised device-measured PA, which is less prone to measurement errors and free of self-reporting bias compared to PA questionnaires. This study also ascertained affective disorders from multiple electronic health records, thus improving data completeness and protecting against reporting bias. To minimise reverse causation due to sub/pre-clinical mental health problems, we conducted a landmark analysis, excluding participants who were diagnosed with affective disorders within the first 2 years of follow-up. BMI and longstanding illnesses could be mediators or confounders and were additionally adjusted in a sensitivity analysis. All of the results were consistent, increasing confidence in the findings. However, this study still has several caveats. Firstly, UK Biobank is not representative of the UK population, with evidence of a healthy volunteer selection bias. Estimates of effect size were found to be consistent with population-representative cohorts [26], but PA levels are higher among UK Biobank participants. Participants with worse mental health might not be recruited or willing to participate from the accelerometer measurements. Therefore, the calculated PFPs are likely to be an underestimate of the contribution of PA to affective disorders in the general population. PFPs assume the estimates to be causal which cannot be confirmed in this study. Even though using clinical diagnosis in linked primary care and hospital data should be relatively robust, it is not possible to differentiate the severity of the mental health outcomes. In spite of adjustment for a wide range of potential confounders, unobserved and residual confounding cannot be ruled out. Reverse causation due to sub-/pre-clinical mental health symptoms is still possible despite the 2-year landmark analysis. Additionally, the wrist-worn accelerometers could overestimate PA levels and may not differentiate light PA from sedentary time as well as in waist-worn accelerometers. Last but not least, even though PA were categorised by intensity, variations within categories could not be captured, and the number and duration of bouts have not been explored.

Even though causality cannot be confirmed in this observational study, the relationship between PA and affective disorders has met most of the Branford Hill criteria for causation [27]. This study has shown the associations to be strong in magnitude (HR < 0.5) with evidence of a dose-response relationship. The associations between PA and lower risk of affective disorders were consistent with previous meta-analyses [6, 7]. There is experimental evidence, including multiple trials, supporting the protective effects of PA on mental well-being across all age ranges [9, 10]. A bidirectional Mendelian randomisation (MR) study also supported a causal relationship between device-measured PA and lower risk of depression, but not the other way round [28]. Interestingly, there was also no association between self-reported PA and depression in the MR study, highlighting the importance of objectively measured PA on reducing recall bias and regression dilution bias. The causal relationship between PA and affective disorders is biologically plausible given the observed changes in several neurotransmitters (including endorphin) after PA [29]. There are several potential mechanisms between PA and affective disorders including neurobiological and psychosocial pathways [30, 31]. The differential associations of VPA with depression and anxiety indicate that there might be different mechanistic pathways, as shown in studies on the signalling of serotonin, norepinephrine, and dopamine [32]. It should be noted that even though similar relationships have been reported in previous meta-analyses and randomised controlled trials [9, 10], the current study adds meaningful evidence through its comparison between device-measured PA of different intensities, examination of dose-response relationships, and consideration of both individual- and population-level implications. However, due to the nature of accelerometer data collection, this study was not able to distinguish between occupational and leisure time PA and its associations with the mental health outcomes of interest.

There have been limited studies comparing PA of different intensities, especially LPA. The study found no significant association between LPA and the affective disorders regardless of adjustment of MPA and VPA. This may indicate that intensity does play an important role in the benefits of PA in mental health. This is indeed true as shown in the linear analysis that for an equivalent amount of time, VPA, compared with MPA, had a stronger association with affective disorder. A cross-sectional study reported slightly stronger effect size for self-reported VPA (OR = 0.58–0.78) than MPA (OR = 0.68–0.92) [33]. However, the PA variables were dichotomised based on a previous recommendation from the American Heart Association (AHA) (MPA ≥ 150; VPA ≥ 60 min/week) [34]. The World Health Organization and the AHA have subsequently updated their recommendation to at least 150–300 or 75–150 min/week of MPA and VPA, respectively [35]. Our study, using the updated recommendation, found that the effect size for achieving the recommendations was slightly weaker for VPA (HR = 0.68) than for MPA (HR = 0.55), even though the confidence intervals overlapped substantially. It should also be noted that since the majority of the participants who performed 75–150 min/week of VPA already were performing a high level of MPA, the potential benefits of VPA estimated could be plateaued.

Only 5.2% of all the affective disorders, including depression and anxiety, could be attributable to not achieving the recommendation of MPA, but 16.5% could be attributable to that of VPA. This is largely due to the substantially different distributions of MPA and VPA. A large majority (95.9%) of study participants fulfilled the WHO recommendation of ≥150 min of MPA (including walking at a moderate speed [36]) but only 9.6% of the participants had ≥75 min of VPA, which could be because the WHO recommendation was largely derived from studies using self-reported PA considering only bouted (e.g. >10 min) PA, and/or that the UK Biobank participants had healthier lifestyle. Regardless, to our knowledge, this is a novel finding as previous studies often considered combined moderate- and vigorous-intensity physical activities rather than MPA, and VPA separately. It is also worth to note that more affective disorders could have been prevented if more participants could perform more MPA than the current recommendations. Future studies should investigate how the current recommendations based on self-reported data should be translated to device-measured PA, given that wearable devices reporting activity time are increasingly common.

The current study, along with previous evidence on causality [9, 28], suggested that promoting MPA and VPA in the population could substantially reduce the burden of affective disorders. Because the associations did not plateau until a very high level of MPA, people who are physically active could still gain benefit from increasing their MPA, echoing the current WHO recommendation. For people who already performed a reasonable amount of MPA (>300 min/week) or for people who would like to achieve more benefits for the same amount of time, they should consider undertaking or increasing VPA. At the population level, strategies to promote VPA should be explored because most people performed minimal VPA. Nonetheless, it should be noted that PA with very high intensity could be associated with discomfort and might not be easy to adopt or maintain [37, 38]. There were also reports on VPA-associated adverse events in trials among older adults [39, 40]. There were not sufficient numbers of participants with high level of VPA but low level of MPA to explore such activity pattern is associated with mental health benefits. Similarly, it is not certain whether performing VPA over the current recommendations of 75–150 min/week would confer to mental health benefits.

## Conclusions

In conclusion, device-measured PA was associated with lower risk of affective disorders. Promoting physical activity in the population, even beyond the current recommendations, is associated with substantially lower burden of affective disorders.

## Supplementary Information

**Additional file 1: Table S1**. Participant characteristics by LPA quartiles. **Table S2**. Participant characteristics by MPA quartiles. **Table S3**. Participant characteristics by VPA quartiles. **Figure S1**. Hypothesised causal diagram. **Figure S2**. Participant flowchart. **Figure S3**. The associations between device-measured PA and affective disorders without adjusting for other intensity-specific PA. **Figure S4**. The associations between device-measured PA and affective disorders adjusting for BMI and longstanding illnesses. **Figure S5**. Two-year landmark analysis for the associations between device-measured PA and affective disorders.

## Data Availability

Data can be requested from UK Biobank (http://ukbiobank.ac.uk/).

## Abbreviations

BMI: Body mass index
CI: Confidence interval
HR: Hazard ratio
ICD10: International Classification of Diseases, 10th revision
IQR: Interquartile range
LPA: Light-intensity physical activity
MET: Metabolic equivalent of task
*mg*: Milligravity
MPA: Moderate-intensity physical activity
PA: Physical activity
PFP: Preventable fraction for the population
SD: Standard deviation
VPA: Vigorous-intensity physical activity
