# Supplementary material for: Device-measured physical activity and incident affective disorders
Source: BMC Med. 2022 Sep 6;20:290. doi: 10.1186/s12916-022-02484-0 (PMC9446787; doi:10.1186/s12916-022-02484-0)
Supplement: Supplementary file 1 — Additional file 1: Table S1. Participant characteristics by LPA quartiles. Table S2. Participant characteristics by MPA quartiles. Table S3. Participant characteristics by VPA quartiles. Figure S1. Hypothesised causal diagram. Figure S2. Participant flowchart. Figure S3. The associations between device-measured PA and affective disorders without adjusting for other intensity-specific PA. Figure S4. The associations between device-measured PA and affective disorders adjusting for BMI and longstanding illnesses. Figure S5. Two-year landmark analysis for the associations between device-measured PA and affective disorders. [file 12916_2022_2484_MOESM1_ESM.docx]

Online-only Supplement for

Device-measured physical activity and incident affective disorders

Frederick K Ho^1^, PhD, Fanny Petermann-Rocha^1,2,3^, MSc, Solange Parra-Soto^1,2^, MSc, Jirapitcha Boonpor^2^, MSc, Jason M R Gill^2^, PhD, Stuart R Gray^2^, PhD,
Jill P Pell^1*^, MD, Carlos Celis-Morales^2,4*^, PhD

1. Institute of Health and Wellbeing, University of Glasgow, Glasgow, United Kingdom
2. Institute of Cardiovascular & Medical Sciences, University of Glasgow, Glasgow, United Kingdom
3. Facultad de Medicina, Universidad Diego Portales, Santiago, Chile.
4. Human Performance Lab, Education, Physical Activity and Health Research Unit, University Católica del Maule, Talca, 3466706, Chile.

*Joint senior author

Corresponding author:

Dr Carlos Celis-Morales

Institute of Cardiovascular and Medical Sciences

University of Glasgow

Glasgow, G12 8TA

United Kingdom

Tel: + 44 141 3304201

Email: [Carlos.Celis@glasgow.ac.uk](mailto:Carlos.Celis@glasgow.ac.uk)

[**Table S1**. Participant characteristics by LPA quartiles 2](#_Toc108530213)

[**Table S2**. Participant characteristics by MPA quartiles 3](#_Toc108530214)

[**Table S3**. Participant characteristics by VPA quartiles 4](#_Toc108530215)

[**Figure S1**. Hypothesised causal diagram 5](#_Toc108530216)

[**Figure S2**. Participant flowchart 6](#_Toc108530217)

[**Figure S3**. The associations between device-measured PA and affective disorders without adjusting for other intensity-specific PA 7](#_Toc108530218)

[**Figure S4**. The associations between device-measured PA and affective disorders adjusting for BMI and longstanding illnesses 8](#_Toc108530219)

[**Figure S5**. Two-year landmark analysis for the associations between device-measured PA and affective disorders 9](#_Toc108530220)

# **Table S1**. Participant characteristics by LPA quartiles

|  | **Overall** | **Device-measured LPA, minutes/week** | | | |
| --- | --- | --- | --- | --- | --- |
|  |  | **≤1754** | **>1754 to 2046** | **>2046 to 2339** | **>2339** |
| Total n | 37237 | 9294 | 9480 | 9243 | 9220 |
| Age, years, mean (SD) | 56.41 (7.76) | 58.65 (7.38) | 56.98 (7.67) | 55.76 (7.70) | 54.32 (7.64) |
| Male | 16907 (45.4) | 4999 (54.9) | 4264 (45.7) | 4020 (42.6) | 3624 (38.7) |
| Non-White ethnicity | 1042 ( 2.8) | 244 ( 2.7) | 242 ( 2.6) | 246 ( 2.6) | 310 ( 3.3) |
| Deprivation index, mean (SD) | -1.84 (2.71) | -1.65 (2.83) | -1.88 (2.69) | -1.95 (2.63) | -1.86 (2.68) |
| College or University degree | 16103 (43.2) | 3715 (40.8) | 4159 (44.5) | 4226 (44.8) | 4003 (42.7) |
| PA volume^a^, mg, mean (SD) | 28.00 (7.82) | 19.07 (3.10) | 24.97 (2.24) | 29.73 (2.96) | 37.98 (5.51) |
| LPA, minutes/week, mean (SD) | 2052.94 (434.05) | 1588.98 (272.07) | 1955.66 (248.11) | 2185.81 (284.27) | 2467.46 (355.04) |
| MPA, minutes/week, mean (SD) | 483.34 (233.67) | 251.44 (102.00) | 395.58 (105.36) | 520.75 (122.31) | 758.76 (208.86) |
| VPA, minutes/week, mean (SD) | 31.48 (37.79) | 10.96 (15.23) | 21.56 (23.48) | 33.11 (32.28) | 59.70 (50.47) |
| Sleep duration, hours/day, mean (SD) | 7.18 (0.96) | 7.24 (1.04) | 7.19 (0.95) | 7.17 (0.93) | 7.12 (0.90) |
| Smoking |  |  |  |  |  |
| Never | 21642 (58.1) | 4886 (53.6) | 5448 (58.3) | 5610 (59.5) | 5698 (60.9) |
| Previous | 13237 (35.5) | 3469 (38.1) | 3317 (35.5) | 3301 (35.0) | 3150 (33.6) |
| Current | 2358 ( 6.3) | 753 ( 8.3) | 572 ( 6.1) | 517 ( 5.5) | 516 ( 5.5) |
| Alcohol intake, units/week, mean (SD) | 15.98 (16.00) | 16.24 (17.11) | 16.22 (16.30) | 15.80 (15.14) | 15.65 (15.42) |
| Fruits/vegetable intake, portions/week, mean (SD) | 4.21 (2.25) | 3.93 (2.16) | 4.10 (2.14) | 4.25 (2.23) | 4.53 (2.41) |
| Red meat intake, portions/week, mean (SD) | 2.06 (1.38) | 2.18 (1.40) | 2.09 (1.38) | 2.02 (1.34) | 1.95 (1.38) |
| Processed meat intake, times/week, mean (SD) | 2.80 (1.05) | 2.91 (1.05) | 2.81 (1.04) | 2.77 (1.05) | 2.71 (1.07) |
| Oily fish intake, times/week, mean (SD) | 2.66 (0.90) | 2.65 (0.89) | 2.67 (0.90) | 2.66 (0.88) | 2.65 (0.91) |
| BMI, kg/m^2^, mean (SD) | 26.70 (4.44) | 28.37 (5.04) | 26.94 (4.27) | 26.25 (4.00) | 25.28 (3.79) |
| Longstanding illnesses | 10137 (27.2) | 3359 (36.9) | 2610 (28.0) | 2203 (23.4) | 1965 (21.0) |

# **Table S2**. Participant characteristics by MPA quartiles

|  | **Overall** | **Device-measured MPA, minutes/week** | | | |
| --- | --- | --- | --- | --- | --- |
|  |  | **≤313** | **>313 to 444** | **>444 to 615** | **>615** |
| Total n | 37237 | 9409 | 9040 | 9599 | 9189 |
| Age, years, mean (SD) | 56.41 (7.76) | 59.62 (6.91) | 56.87 (7.66) | 55.29 (7.69) | 53.84 (7.55) |
| Male | 16907 (45.4) | 4602 (48.9) | 4167 (46.1) | 4315 (45.0) | 3823 (41.6) |
| Non-White ethnicity | 1042 ( 2.8) | 219 ( 2.3) | 250 ( 2.8) | 280 ( 2.9) | 293 ( 3.2) |
| Deprivation index, mean (SD) | -1.84 (2.71) | -1.75 (2.77) | -1.94 (2.64) | -1.88 (2.69) | -1.77 (2.72) |
| College or University degree | 16103 (43.2) | 3621 (38.5) | 3930 (43.5) | 4445 (46.3) | 4107 (44.7) |
| PA volume^a^, mg, mean (SD) | 28.00 (7.82) | 19.71 (3.85) | 25.30 (3.65) | 29.72 (4.12) | 37.36 (5.79) |
| LPA, minutes/week, mean (SD) | 2052.94 (434.05) | 1794.06 (408.28) | 2021.04 (392.15) | 2125.83 (389.04) | 2273.28 (399.62) |
| MPA, minutes/week, mean (SD) | 483.34 (233.67) | 222.72 ( 68.74) | 383.51 ( 37.53) | 526.72 ( 48.21) | 803.11 (170.41) |
| VPA, minutes/week, mean (SD) | 31.48 (37.79) | 10.45 (19.30) | 23.48 (29.32) | 35.55 (35.44) | 56.64 (45.62) |
| Sleep duration, hours/day, mean (SD) | 7.18 (0.96) | 7.22 (1.05) | 7.18 (0.95) | 7.17 (0.93) | 7.15 (0.89) |
| Smoking | |  |  |  |  |
| Never | 21642 (58.1) | 5012 (53.3) | 5303 (58.7) | 5697 (59.3) | 5630 (61.3) |
| Previous | 13237 (35.5) | 3621 (38.5) | 3182 (35.2) | 3368 (35.1) | 3066 (33.4) |
| Current | 2358 ( 6.3) | 776 ( 8.2) | 555 ( 6.1) | 534 ( 5.6) | 493 ( 5.4) |
| Alcohol intake, units/week, mean (SD) | 15.98 (16.00) | 15.57 (16.72) | 15.92 (16.06) | 16.12 (15.49) | 16.29 (15.72) |
| Fruits/vegetable intake, portions/week, mean (SD) | 4.21 (2.25) | 4.05 (2.20) | 4.09 (2.15) | 4.23 (2.23) | 4.46 (2.38) |
| Red meat intake, portions/week, mean (SD) | 2.06 (1.38) | 2.19 (1.41) | 2.09 (1.34) | 2.03 (1.38) | 1.92 (1.36) |
| Processed meat intake, times/week, mean (SD) | 2.80 (1.05) | 2.90 (1.04) | 2.82 (1.04) | 2.78 (1.05) | 2.70 (1.08) |
| Oily fish intake, times/week, mean (SD) | 2.66 (0.90) | 2.68 (0.89) | 2.67 (0.89) | 2.65 (0.89) | 2.62 (0.91) |
| BMI, kg/m^2^, mean (SD) | 26.70 (4.44) | 28.45 (5.09) | 26.86 (4.20) | 26.22 (3.99) | 25.24 (3.72) |
| Longstanding illnesses | 10137 (27.2) | 3575 (38.0) | 2487 (27.5) | 2230 (23.2) | 1845 (20.1) |

# **Table S3**. Participant characteristics by VPA quartiles

|  | **Overall** | **Device-measured VPA, minutes/week** | | | |
| --- | --- | --- | --- | --- | --- |
|  |  | **≤10** | **>10 to 20** | **>20 to 40** | **>40** |
| Total n | 37237 | 15856 | 6717 | 6903 | 7761 |
| Age, years, mean (SD) | 56.41 (7.76) | 58.55 (7.27) | 56.41 (7.53) | 55.33 (7.63) | 53.01 (7.63) |
| Male | 16907 (45.4) | 6321 (39.9) | 3107 (46.3) | 3394 (49.2) | 4085 (52.6) |
| Non-White ethnicity | 1042 ( 2.8) | 381 ( 2.4) | 178 ( 2.6) | 203 ( 2.9) | 280 ( 3.6) |
| Deprivation index, mean (SD) | -1.84 (2.71) | -1.71 (2.77) | -1.88 (2.72) | -1.99 (2.60) | -1.91 (2.65) |
| College or University degree | 16103 (43.2) | 6495 (41.0) | 2885 (43.0) | 2942 (42.6) | 3781 (48.7) |
| PA volume^a^, mg, mean (SD) | 28.00 (7.82) | 22.76 (5.14) | 27.54 (5.03) | 30.47 (5.40) | 36.92 (7.08) |
| LPA, minutes/week, mean (SD) | 2052.94 (434.05) | 1963.35 (445.73) | 2096.26 (418.55) | 2133.88 (411.44) | 2126.51 (408.23) |
| MPA, minutes/week, mean (SD) | 483.34 (233.67) | 341.19 (161.59) | 491.74 (172.14) | 575.99 (193.13) | 684.10 (246.04) |
| VPA, minutes/week, mean (SD) | 31.48 (37.79) | 6.61 ( 4.79) | 20.16 ( 0.00) | 34.24 ( 4.93) | 89.66 (44.90) |
| Sleep duration, hours/day, mean (SD) | 7.18 (0.96) | 7.20 (1.00) | 7.17 (0.96) | 7.17 (0.93) | 7.15 (0.87) |
| Smoking | |  |  |  |  |
| Never | 21642 (58.1) | 8801 (55.5) | 3869 (57.6) | 4072 (59.0) | 4900 (63.1) |
| Previous | 13237 (35.5) | 5899 (37.2) | 2423 (36.1) | 2411 (34.9) | 2504 (32.3) |
| Current | 2358 ( 6.3) | 1156 ( 7.3) | 425 ( 6.3) | 420 ( 6.1) | 357 ( 4.6) |
| Alcohol intake, units/week, mean (SD) | 15.98 (16.00) | 15.07 (16.02) | 16.35 (15.96) | 16.92 (16.58) | 16.66 (15.37) |
| Fruits/vegetable intake, portions/week, mean (SD) | 4.21 (2.25) | 4.15 (2.20) | 4.19 (2.24) | 4.18 (2.22) | 4.36 (2.37) |
| Red meat intake, portions/week, mean (SD) | 2.06 (1.38) | 2.12 (1.39) | 2.10 (1.39) | 2.03 (1.37) | 1.92 (1.32) |
| Processed meat intake, times/week, mean (SD) | 2.80 (1.05) | 2.82 (1.04) | 2.82 (1.05) | 2.79 (1.06) | 2.76 (1.08) |
| Oily fish intake, times/week, mean (SD) | 2.66 (0.90) | 2.68 (0.89) | 2.66 (0.88) | 2.64 (0.90) | 2.62 (0.91) |
| BMI, kg/m^2^, mean (SD) | 26.70 (4.44) | 27.70 (4.90) | 26.62 (4.13) | 26.09 (3.90) | 25.28 (3.60) |
| Longstanding illnesses | 10137 (27.2) | 5434 (34.3) | 1782 (26.5) | 1560 (22.6) | 1361 (17.5) |

# **Figure S1**. Hypothesised causal diagram

Physical activity

Affective disorder

Sociodemographic factors

(age, sex, ethnicity, deprivation, education)

Other lifestyle factors

(sleep, smoking, alcohol and dietary intake)

BMI

Longstanding illnesses

Possible mediators or confounders

(Sensitivity analysis)

Some causal pathways (e.g. from sociodemographic factors to affective disorders) were omitted for clarity.

# **Figure S2**. Participant flowchart

All UK Biobank participants

N = 502,459

N = 96,519

Excluded 405,963 participants without valid device-measured PA data

N = 43,039

Excluded 53,480 participants without linked primary care data

Excluded 5712 participants with prior affective disorders

Included in analyses:

N=37,327 for primary analysis

N=36,532 for two-year landmark analysis

# **Figure S3**. The associations between device-measured PA and affective disorders without adjusting for other intensity-specific PA


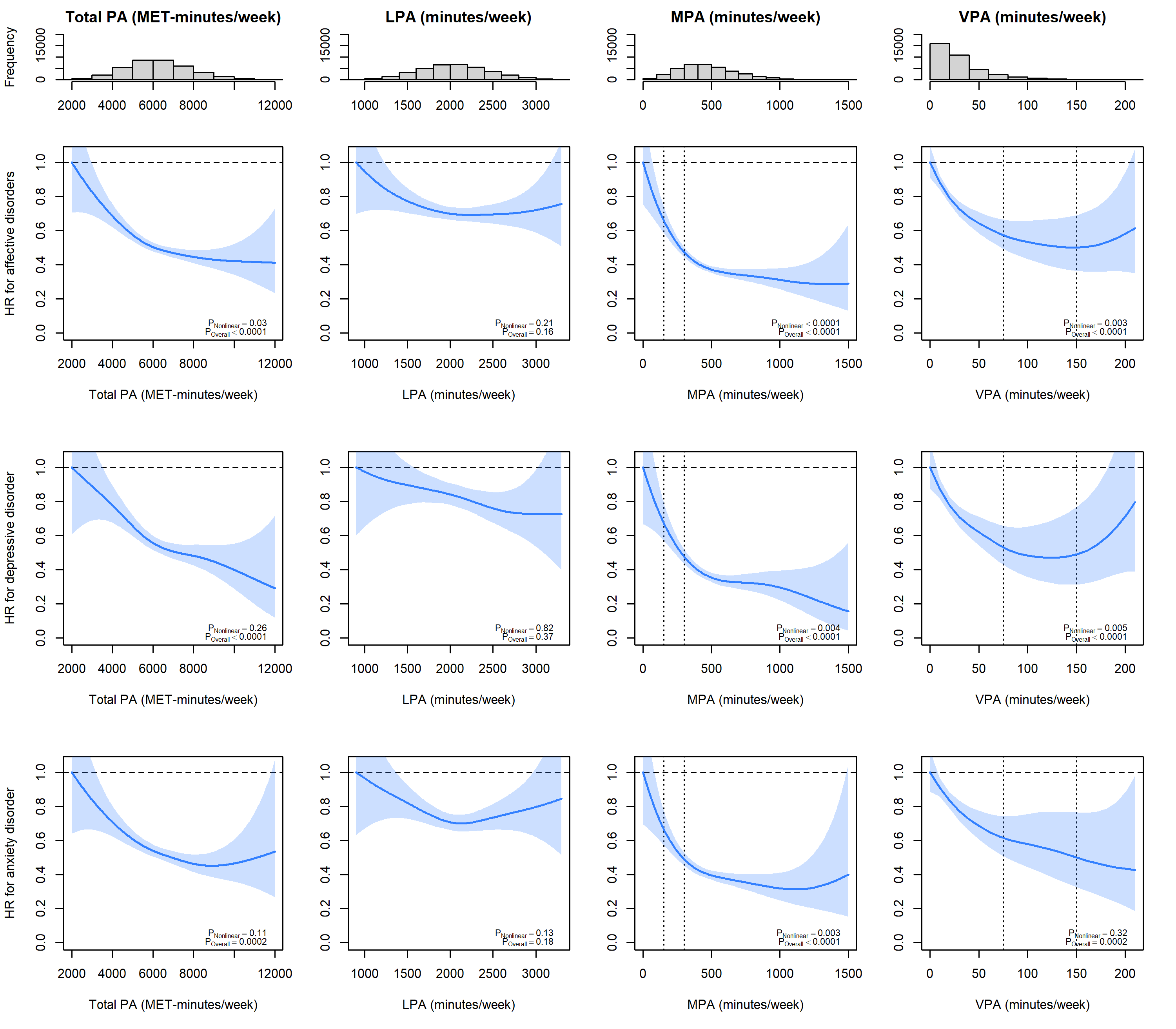


All adjusted for age, sex, ethnicity, education, deprivation index, sleep duration, smoking, alcohol intake, dietary intake of fruits/vegetables, red meat, processed meat, and oily fish

Vertical dashed lines represent current WHO recommendations.

LPA: light intensity PA; MPA: moderate intensity PA; VPA; vigorous intensity PA

# **Figure S4**. The associations between device-measured PA and affective disorders adjusting for BMI and longstanding illnesses


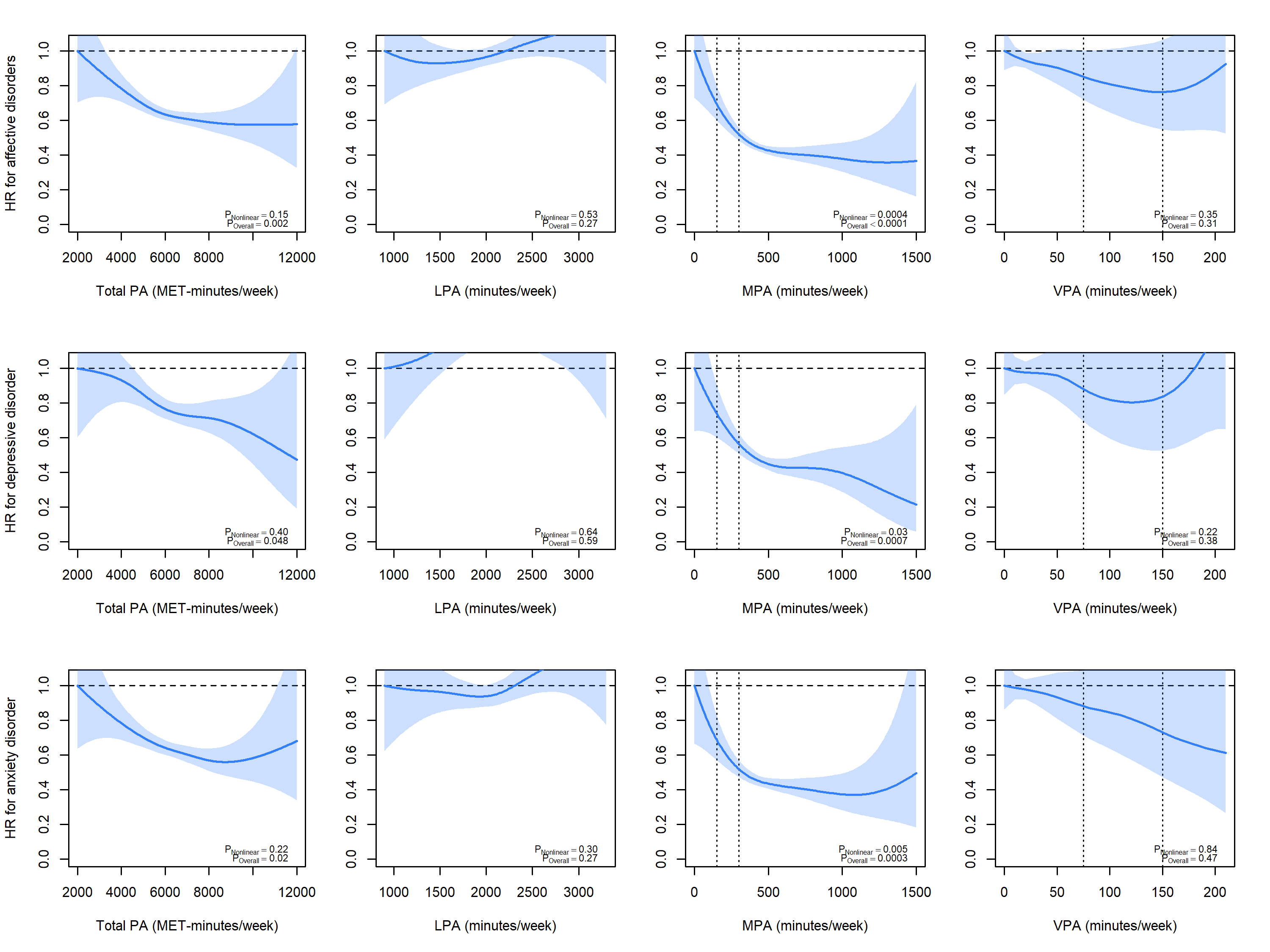


LPA, MPA and VPA were mutually adjusted; all adjusted for age, sex, ethnicity, education, deprivation index, sleep duration, smoking, alcohol intake, dietary intake of fruits/vegetables, red meat, processed meat, and oily fish, BMI, and longstanding illnesses

Vertical dashed lines represent current WHO recommendations.

LPA: light intensity PA; MPA: moderate intensity PA; VPA; vigorous intensity PA

# **Figure S5**. Two-year landmark analysis for the associations between device-measured PA and affective disorders


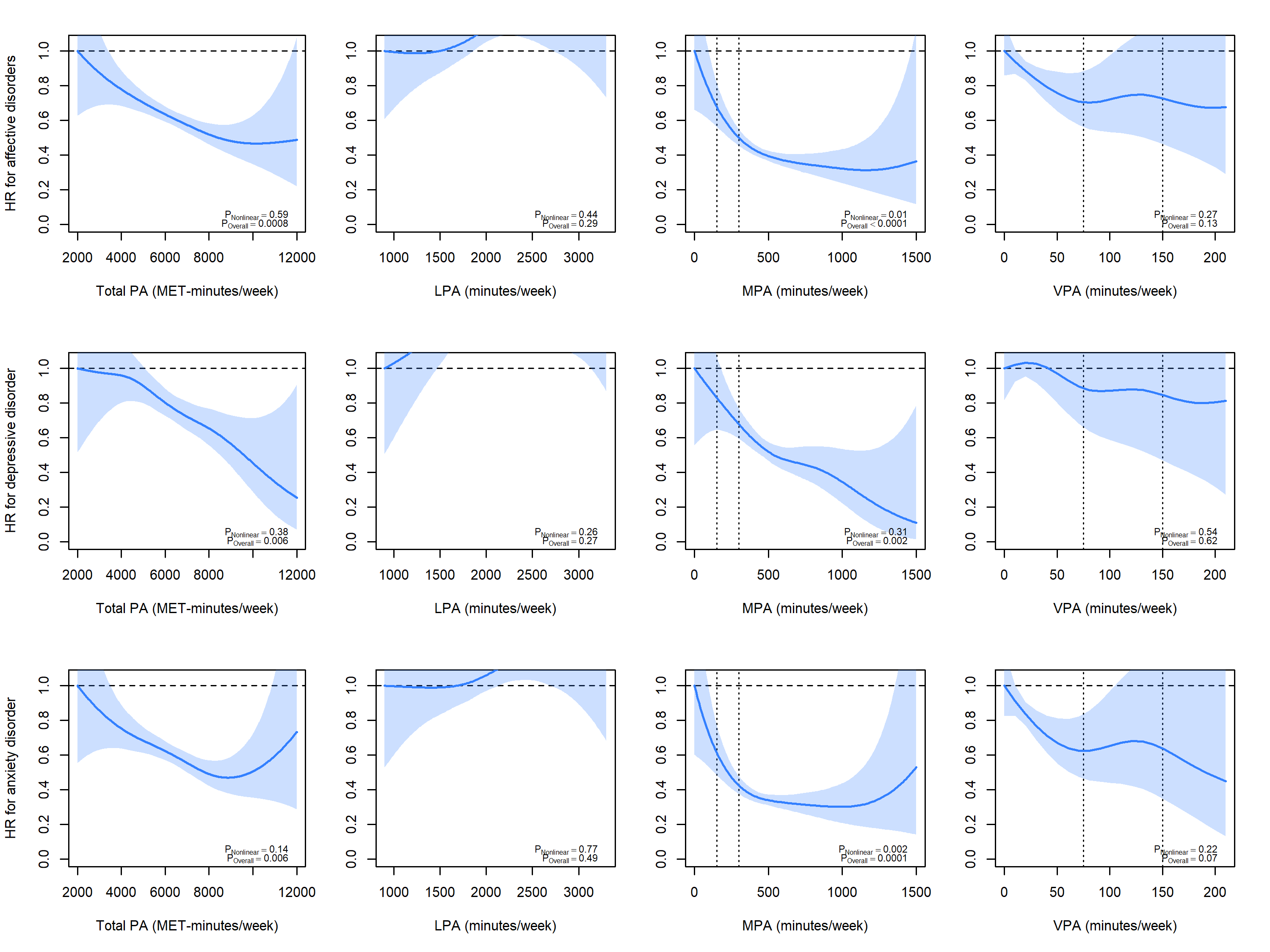


Participants who had outcomes in the first two years of follow-up were excluded.

LPA, MPA and VPA were mutually adjusted; all adjusted for age, sex, ethnicity, education, deprivation index, smoking, alcohol intake, dietary intake of fruits/vegetables, red meat, processed meat, and oily fish.

Vertical dashed lines represent current WHO recommendations.

LPA: light intensity PA; MPA: moderate intensity PA; VPA; vigorous intensity PA
